# Gaps in Medical Students' Competencies to Deal With Intimate Partner Violence in Key Mozambican Medical Schools

**DOI:** 10.3389/fpubh.2019.00204

**Published:** 2019-07-24

**Authors:** Beatriz Manuel, Kristien Roelens, Armindo Tiago, Ines Keygnaert, Martin Valcke

**Affiliations:** ^1^Department of Community Health, Faculty of Medicine, University Eduardo Mondlane, Maputo, Mozambique; ^2^Department of Public Health and Primary Care, International Centre for Reproductive Health, Ghent University, Ghent, Belgium; ^3^Department of Obstetrics and Gynecology, Faculty of Medicine and Health Sciences, Ghent University, Ghent University Hospital, Ghent, Belgium; ^4^Department of Physiology, Faculty of Medicine, University Eduardo Mondlane, Maputo, Mozambique; ^5^Department of Educational Studies, Ghent University, Ghent, Belgium

**Keywords:** intimate partner violence, medical education, medical students, competencies, Mozambican medical curricula

## Abstract

**Purpose:** The researchers aimed to identify the gaps in competencies designed to help medical students to deal with Intimate Partner Violence (IPV) in key Mozambican medical schools curricula.

**Method:** A survey was administered to 3rd and 6th-year medical students (N387), enrolled in five medical schools in Mozambique. The instrument focused on mapping students' perceived mastery of their knowledge, skills, and attitudes related to IPV.

**Results:** In total, 387 medical students (RR 66%) participated in the survey. The overall mean perceived mastery of IPV competence was 36.18 (SD = 24.52) for knowledge, 32.01 (SD = 27.37) for skills, and 43.47 (SD = 27.58) for attitudes. Though 6th-year students reported a significantly higher mastery level, it is still below a mastery-learning benchmark of 80%.

**Conclusions:** Medical students report critically low levels in their mastery of IPV- related competencies. This implies a need for a more comprehensive approach to developing knowledge, skills, and attitudes to deal with the victims of IPV.

## Introduction

Intimate Partner Violence (IPV) focuses on violence committed in a present or past relationship. IPV is an enduring problem in Sub-Saharan Africa (SSA). However, are medical doctors adequately trained to understand and cope with IPV? In this article, we focus on IPV prevalence in SSA. This introduces the setting to analyze students' perceived mastery of IPV competencies aligned with recognizing and dealing with IPV in Mozambican medical schools.

### Worldwide Intimate Partner Violence Prevalence, Incidence

Intimate Partner Violence, also known as *battering, domestic violence, family violence, gender-based violence, partner abuse, partner violence, family violence, abuse, spouse abuse, and wife assault*, is a critical public health issue. The World Health Organization (WHO) defines *Intimate Partner Violence* as “any behavior within an intimate relationship that leads to physical, psychological or sexual harm to those in that relationship” (1). As stated by the WHO (2), “unintended pregnancies, abortions, adverse pregnancy and neonatal and infant outcomes, sexually transmitted infections (including HIV) and mental disorders (such as depression, anxiety disorders, sleep disorders, and eating disorders)” are reflected to a greater extent in abused women than in non-abused women. Zacarias (3) stressed that males can also be victims of IPV.

In SSA, only 10% of all cases of IPV are reported to the police (4). Many women in SSA are forced through violence to maintain sexual relations with their partners, by means of violence and cannot negotiate the use of condoms leading to vulnerability to HIV (5). Cruz et al. (4) added that in official and traditional marriage or in sexual unions where patriarchal views and gender roles prevail, women are marginalized and often seen as the property of men.

Few research reports of exclusive violence against men perpetrated by a female partner in SSA (6). The same authors stated that in 2012, journalists from a local news in the Nyeri region of Kenya, have reported that women justify violence against their male partners “by impugning their husbands' drinking or failure to sustain employment, in other words, for failing to uphold male gender roles in marriage mirroring the excuses given for beating wives” (p. 283). In a study conducted in Dar es Salaam, Tanzania, the authors found that within 2017, 55.3% of men and 33.3% of women reported perpetrating IPV within the same time period and from those, 30.6% of male perpetrators and 54.6% of female perpetrators also reported physical IPV victimization (7). A national household surveys of persons aged 13–24 years to measure experiences of violence victimization in childhood and subsequent perpetration of physical or sexual violence conducted by Swedo et al. [(8), p. 350], in Malawi, Nigeria, Uganda, and Zambia, reported that perpetration of physical or sexual violence was prevalent among both males and females, ranging among males from 29.5% in Nigeria to 51.5% in Malawi and among females from 15.3% in Zambia to 28.4% in Uganda.

### Intimate Partner Violence in Mozambique

The above also applies to Mozambique due to patriarchal sociocultural beliefs, poor implementation of equity and empowerment strategies and lack of gender mainstreaming (9). In Mozambique, more than 29,000 women mentioned they are a survivor of violence, an average of more than 7,000 women per year (10). Since many do not report abuse, because of stigmatization, or cultural, social, and economic reasons, real numbers are probably much higher. Misconceptions and lack of information about IPV lead to a reality that “one-third of women believe that a husband is justified in beating his wife” (11).

In the last Demographic Health Survey (DHS) from Mozambique one of three Mozambican women, aged 15–49, reported having been a survivor of physical violence since the age of 15, with the husband or intimate partner as the perpetrator in 62% of the cases (12). Moreover, 12% of all women reported having suffered sexual violence since the age of 15. In general, 50% of reported cases of violence against women were related to sexual abuse (11). The last DHS from Mozambique revealed that 32% of women reported being victims of IPV, compared to 12% in men. In total, 26% of women and 8% of men reported having been victims of IPV during the last 12 months of the last DHS.

A high prevalence of IPV against men is observed in the province of Cabo Delgado, north of Mozambique (12): 28% of men reported being victims of IPV and 20% reported having suffered IPV during the last 12 months of the last DHS (12).

Many families prefer to deal with IPV using traditional justice systems, particularly in rural communities. Moreover, Zacarias et al. (13) stated that only three studies did address the prevalence of IPV against men in Mozambique (12, 14, 15). Most survivors do not report IPV because “the victim can deal with it alone or through the extended family” (47%), the violent act is considered “not serious” (15%) or as a “private issue” (9%), or due to “fear of retaliation from the perpetrator” (11%) [(4), p. 1,591].

### Intimate Partner Violence and Health Care Providers

IPV is under-recognized by health care providers (16–20). These authors refer to a reluctance to deal with the issue for fear of retaliation, police involvement, time constraints, cultural differences, lack of trust, lack of knowledge, skills, confidence, limited resources to manage IPV, limited referral points, conflicting cultural and gender norms, or discomfort with the subject. In the Mozambican context, non-disclosure of violence against men is mostly linked to the lack of privacy in attendance. Most “spaces” attending to victims of violence tend to be “feminized” (21). As reported by Swailes et al. (22) given the sensitive nature of IPV-related medical-patient communication, the provider's initial response has potential to influence a patient's course of action once he/she leaves the consultation room. The same authors added that disclosing IPV warrants direct in-depth communication. The provider's initial response should focus on validation of the patient's feelings, therefore establishing a way of empathy with the victim. More discussion should encompass assessing the safety of the victim and family members, the pattern and severity of abuse and embody a safety set- up if abusive behavior escalates. Likewise, the provider also has to assess the impact IPV has on the survivor's physical health, mental state and social relationships to understand the approach for more dialogue and intervention (22).

### IPV in Medical Education

Since survivors of IPV often have the first contact with a primary care medical doctor, this may be a key opportunity to tackle IPV efficiently and effectively and in a non-judgmental way (23). In sub-Saharan settings, as there is a lack of medical doctors, it happens that the first contact with the victims of IPV is with professionals at a lower level. Accordingly to Colarossi et al. (24) “family planning service providers are the most likely ones to see women during the years that coincide with the highest risk of victimization” (p. 236). However, medical doctors seem reluctant to ask about abuse issues. In other cases, they do not respond appropriately, mostly due to lack of expertise, training, time constraints, and lack of resources (23, 25). Lack of training on IPV may lead to absence of screening for IPV, lack of confidence when dealing with victims of IPV, minimal knowledge about referral points and little or no intervention (26–29).

IPV competencies combine a complex of knowledge, attitude, and skill components. Based on a literature review, we used an IPV curriculum framework for medical students, mainly based on IPV literature and available guidelines (19, 30–39). This competence framework—reflected in Appendix 1—aims at combining competencies that help ensure medical students can work efficiently with IPV survivors.

When it comes to “knowledge,” students should be able to define IPV, understand the magnitude of the problem and related risk factors and the effect on the survivor, family members and community, next to understanding legal options and reporting requirements. “attitudes” stress students' orientation toward IPV treatment and intervention. Lastly, “skills” refer to identification, documentation and referral strategies.

Little information is known about medical students' comprehensive mastery of these IPV and how this sensitive topic should be taught to acquire better communication skills following screening and patient perceptions of those encounters. Most studies focus on specific components. For instance, Kamimura et al. (40) compared students' opinions, knowledge and attitudes toward IPV in medical curricula of the US, Vietnam China. US students reported higher knowledge levels as compared to Vietnamese and Chinese students. but nevertheless, the scarcely available studies mainly stressed the lack of a consistent focus on IPV in medical curricula. This is e.g., found in the study conducted by Valpied et al. (41) conducted in 18 Australian medical schools. At the pre-vocational secondary education level, students seemed to receive little or no IPV education. Carlson et al. found a lack of IPV- related clinical knowledge. Students question the adequacy of their training due to lack of learning opportunities: “confidence about talking to patients about IPV and talking to patients about IPV would be helpful to increase levels of background and knowledge of IPV” [(42), p. 77].

Related studies also point at background variables playing a role, such as sex, with females being more strongly geared to IPV and knowledgeable about it (40), vs. other studies in which males were reported as being more knowledgeable about IPV (30, 42). Other researchers stressed differences between medical schools (28, 40), or differences linked to year (or age) IPV is tackled (43–47). The latter studies emphasize the need to focus on IPV throughout the curriculum (43–47). However, no studies are available linking comprehensive IPV competency mastery to background variables.

Last, the literature is not clear about a critical benchmark for IPV competence mastery. Most authors state that IPV mastery is not “on par” (30, 40, 41, 48). Benchmarks are defined as: “standards for measurement of performance that can be used for comparison and to identify where needs for improvement exist” [(49), p. 4]. Some authors suggest a 70% benchmark, or state this benchmark has to be defined by the program (50), or that exit level benchmarks are critical but take a lot of time and effort to develop (51). In the present study, we adopted the “mastery learning” benchmark of 80% put forward by Zimmerman and Dibenedetto (52). This benchmark is high but seems adequate for competencies that are critical for patient care and survival.

The study reported in this paper was designed to address the following gaps in research and evidence: “a general bias of published literature toward high-income countries” and “evidence comes predominantly from high-income countries and is focused on response, with more research needed on primary prevention, including in low-income and middle-income countries” (53).

### Objectives and Research Questions

The researchers aimed to map the perceived mastery of IPV competence components in Mozambican medical students by focusing on the following three research questions:

1. What is the perceived mastery of knowledge, skills and attitudes in relation to IPV competencies in Mozambican medical students?
2. Is this mastery level different and how does it vary with students' background?
3. Is the perceived mastery of IPV competencies on a par with the mastery-learning benchmark?

## Materials and Methods

### Research Sample

The study was conducted in five medical schools situated in the South, Center and North of Mozambique. Third- and sixth-year students were randomly selected from each medical school. We expected that competence differences between 3rd- and 6th-year students will occur as IPV- related topics are dealt with in the clinical years. Sample size calculation was based on an expected prevalence of 50% of medical students mastering IPV competencies, with a confidence interval of 95%, α 0.05 and critical value Zα/2 of 1.96. This estimation resulted in *n* of 191 third-year students out of 376 and *n* of 144 of 6th-year students out of 228. A first data collection round resulted in responses from 167 participants (50%). A second data collection was conducted, resulting in an additional response from 220 students (66%). The latter is considered a good response rate (54, 55) A total of 304 students represented 80.85% of the 3rd-year student population and 58 students represented 25.44% of the 6th-year population. The smaller proportion of 6th-year students can be linked to their absence from the university during internships and clinical rotations.

All participants signed an informed consent form after receiving an explanation about the focus of the study, that their input would be kept confidential, that participation was voluntary, and that they could withdraw from the study at any time. The ethical approval was obtained from the Institutional Committee of Bioethics and Health linked to the Faculty of Medicine/Central Hospital of Maputo, Mozambique (registration number CIBS FM & MCH/006/2016). In addition, permission was granted by individual medical schools.

### Research Instrument Design and Validation

A Portuguese language survey (IPV-KSA) was developed, based on the IPV framework presented in Appendix 1. Survey design focused on developing 21 items covering IPV competencies; considering the Mozambique context (see Appendix 1). Students were asked to report their self-efficacy perspective in relation to each item on a scale from 0 to 100 (see Appendix 4). The self-efficacy scale was developed, based on the guidelines of Bandura (56).

A pilot study was conducted, involving 20 3rd -year students selected conveniently from a class of students attending a theoretical-practical session in their medical course training (data not used in subsequent data analysis) of each medical school to assess the feasibility of the IPV_KSA, item relevance and clarity. Students were invited to make comments and to give suggestions. The average administration time was estimated at 20 min.

In view of the validation of the IPV_KSA instrument, data from 50% of the respondents were used for exploratory factor analysis (EFA). Next, the remaining data were used to carry out a confirmatory factor analysis (CFA). Cut off values for CFA-goodness-of-fit indices were based on Hu and Bentler (57) and Jackson et al. (58). A cutoff of 0.90 recommended suggests “authors should indicate the cutoff values for fit measures they intend to use” [(57), p. 19]. The EFA confirmed the three-factor KSA-structure of the scale explaining 72% of the total variance (see Appendices 2, 3). Two knowledge items showed a better fit with the attitudes sub-scale. One attitudes-related item showed a better fit with skills since it referred to “follow-up of patients.” Confirmatory factor analysis (CFA) resulted in acceptable goodness-of-fit indices: *X*^2^ = 436.6, Df = 170, *p* < 0.01, CMIN/Df = 2.57, CFI = 0.93; TLI = 0.91, RMSEA = 0.8 (see Appendix 3). The high co-variances between the three factors confirm the interlinked nature of knowledge, skills and attitudes in IPV competencies. Cronbach's alpha reflects a high to very high reliability for each subscale: Knowledge (eight items, alpha = 0.93), Skills (eight items, alpha = 0.94) and Attitudes (six items, alpha = 0.90).

The survey started with a section focusing on student background variables (age groups, male/female students, medical education school, and year in medical education).

### Procedure

The study took place between July and September of 2016. One researcher visited the students in each specific medical school. After obtaining informed consent, students filled out the IPV_KSA during a regular classroom session, in the presence of the researcher to clarify possible queries.

### Analysis Approach

After developing a general picture on the basis of a descriptive analysis of the research variables, IPV mastery was linked to student-background variables (one-way ANOVA). Next, the perceived mastery level was compared to an external benchmark (80%), using one-sample *t*-tests. Next to *p*-values, we reported effect sizes (Cohen's d) to interpret analysis results, considering the guidelines of Baguley (59): *d* > 0.3 = small effect size; *d* > 0.05 = medium effect size; *d* > 0.8 = large effect size. A *p*-value of *p* < 0.01 was put forward. All analyses were carried out using statistical software SPSS version 24.0.

## Results

After screening, data from 362 students were included in the data analysis (*n* 304 or 80.85% 3rd-year students; *n* 58 or 25.44% 6th-year students). Most respondents were male (*n* 238 or 61%). Considering population parameters, females were, underrepresented (*n* 145 or 37%); sex data from 9 students were missing. The average age was 22.33 years (SD = 3.9).

### Perceived Mastery of the IPV Competencies

Table 1 summarizes sociodemographic aspects. At the same time, the table also shows a focus on student-background variables. Overall, the results point at a weak overall mastery of IPV competencies, in particular in IPV knowledge [*F*_(1)_ = 19.89, *p* < 0.05, *d* = 0.4] and IPV skills [*F*_(1)_ = 21.26, *p* < 0.05, *d* = 0.4], and at the level of the IPV competence components, while suggesting differences related to background variables.

**Table 1:** Socio-demographic characteristics of study participants.

**Socio-demographic characteristics**	***n***	**%**
**AGE, YEARS**		
≤20 years old	139	35.9
21 to 22 years old	125	32.3
≥ 22 years old	123	31.8
Mean age	22.33	
**SEX**		
Female	145	37.5
Male	238	61.5
**YEAR IN MEDICAL SCHOOL**		
3rd	304	84
6th	68	16
**YEAR OF IPV INTRODUCTION**		
1st	1	2.5
2nd	6	15
3rd	7	17.5
4th	8	20
5th	13	32.5
6th	5	12.5

### Perceived IPV Competence Mastery and Background Variables

Students were split up into three age groups: (1) up to 20 years old (*n* 139 or 35.9%), (2) from 21 to 22 years old (*n* 125 or 32.3%), and (3) older than 22 years old (*n* 123 or 31.8%). Age group differences are significant, with older students reflecting significantly higher KSA scores [*F*_(1)_ = 49.36, *p* < 0.01*, d* = 0.3] in all medical schools. There were no differences based on the school year of the students.

Females master significantly higher the IPV knowledge [*F*_(1)_ = 5.33, *p* < 0.05*, d* = 0.01] and skills component [*F*_(1)_ = 10.60, *p* < 0.05, *d* = 0.03]; with small effect sizes in all medical schools. Differences in attitudes were not significantly different.

We observed that students from medical schools E, B and D reflect higher IPV competence mastery as compared to C and A in relation to the knowledge [*F*_(1)_ = 19.89, *p* < 0.01, *d* = 0.17], skills [*F*_(1)_ = 21.26, *p* < 0.01, *d* = 0.18] and attitudes component [*F*_(1)_ = 7.5, *p* < 0.01, *d* = 0.1]. Effect sizes were small, meaning that differences in medical schools are trivial as means do not differ by 0.2 standard deviations even as it is statistically significant.

Third-year medical students reported consistently significantly lower perceived IPV competence mastery as compared to 6th year students in the knowledge [*F*_(1)_ = 61.94, *p* < 0.01, *d* = 0.14], skills [*F*_(1)_ = 89.96, *p* < 0.01, *d* = 0.2], and attitudes components [*F*_(1)_ = 37.76, *p* < 0.01, *d* = 0.1] in all medical schools. Effect sizes were small, meaning that differences in the year of medical curriculum are trivial as means do not differ by 0.2 standard deviations even as it is statistically significant.

From a total of 387 medical students that participated in the study, only *n* 40 (10 or 3%) students reported when they were introduced to IPV contents in their medical curriculum. For that reason, we did not analyze data regarding the year of IPV introduction.

### Perceived Mastery Compared to the Mastery-Learning Benchmark

Perceived mastery levels of medical students were compared to an 80% criterion. The one-sample *t*-test results in Table 2 show consistently that mastery is significantly below the benchmark in all KSA components in IPV competence and Table 3 the comparison of the means of the total IPV scores based on some of the student characteristics.

**Table 2:** One-sample *t*-test results related to perceived IPV competence mastery.

	***Df*^**a**^**	**Mean**	**Mean difference**	***SD*^**b**^**	**One-sample *t*-test**
Overall IPV_KSA score	380	36,18	−43,82	24,52	−34,88^**^
Knowledge	385	33,43	−49,05	24,84	−36,83^**^
Skills	383	32,01	−50,73	27,38	−35,35^**^
Attitudes	384	43,47	−36,53	27,58	−25,98^**^

** p < 0.01;

a Df = degrees of freedom;

b *SD = Standard deviation*.

**Table 3:** Comparison of the mean of the total IPV scores based on some of the student characteristics.

**Characteristics**	**Mean ± SD**
**SEX**	
Female	39,30 (24,65)
Male	34,23 (24,31)
**AGE**	
Age groups ≤20	27,72 (20,51)
Age groups 21 or 22	30,97 (22,14)
Age groups ≥22	51,21 (24,37)
**YEAR IN MEDICAL CURRICULA**	
*n* 304 in 3rd	31,34 (22,55)
*n* 58 in 6th	58,61 (20,74)
**YEAR OF IPV INTRODUCTION (*****n*** **= 39)**	
1st (1)	28,32
2nd (6)	49,80 (21,55)
3rd (7)	47,25 (10,60)
4th (8)	62,80 (15,93)
5th (12)	62,05 (23,11)
6th (5)	67,56 (10,64)
**MEDICAL SCHOOL (*****n*****)**	
Medical school A (150)	28,92 (18,00)
Medical school B (57)	49,65 (28,18)
Medical school C (101)	30,18 (24,32)
Medical school D (56)	45,10 (24,95)
Medical school E (23)	55,72 (22,06)

## Discussion

IPV is a global public health issue, which seems hardly addressed in medical curricula as we found in a previous study on global IPV medical curricula. In this study, perceived mastery of IPV competencies was examined in Mozambican medical students. In relation to IPV competencies, a distinction was made—based on a framework—on IPV-related knowledge, skills and attitudes.

The results are striking: they indicate that, in relation to all IPV competencies, perceived mastery of students is low. This applies to overall IPV competence scores, as well as to the Knowledge, Skills and Attitude sub-scale scores.

Students aged 22 or more reported higher KSA scores on IPV as compared to the younger group of students. In Mozambican context, age matters since, according to academic reports from medical schools, about 1/3 of students enter the level of higher education at a later age and as such may bring their personal and professional experiences into the educational context, which can boost their orientation toward dealing with IPV.

Female medical students reported higher perceived mastery scores in IPV knowledge and skills. This is not in line with Fawole et al. (30) who stated that male students reflect stronger knowledge about violence against women and female students reflect higher attitude scores. Differences can be explained by pointing at the Mozambican context where female students are more acutely aware of IPV. On the other hand, the authors reached their conclusions from studies in settings with high prevalence and incidence of male-to-female IPV in most countries, where almost all programs addressing victims of IPV focus on the male as perpetrators of IPV.

Differences between medical schools could be linked to the nature of the curriculum: one school reflected (a) an innovative problem-based learning/community-based curriculum (*n* = 1), compared to (b) a conventional/community-based curriculum in the other universities (*n* = 4); as found in our previous study on evaluation of Mozambican medical curriculum contents on IPV. However, the results are not in line with curriculum design we found in the mentioned medical schools as found in our submitted to peer review study on IPV curriculum evaluation. Students from three medical schools (one innovative and two conventional/community-based) reported being better prepared to manage IPV victims as compared to students from medical schools with conventional curricula. To explain this, we need to look at differences between third-and 6th-year students. As will become clear, despite medical curricula being different, all curricula reflect a lack of a consistent and continuous IPV focus.

As expected, perceived mastery of 3rd-year students is more restricted compared to 6th-year students. This seems aligned with the literature stressing IPV contents should be introduced in preclinical years but developed fully throughout clinical years. However, our findings reinforce the view that few medical schools seem to address IPV consistently (30, 43, 45, 60). Perceived mastery is—even in 6th-year students—not on a par with the 80% benchmark, suggesting this consistent and continuous focus on IPV in the curriculum is lacking. Though the guidelines of the National Action Plan for Prevention and Eradication of Violence Against Women of Mozambique suggest integrating consistently and continuously issues of violence, domestic violence and gender in the curriculum of medical schools, this does not seem to be the case (46, 61, 62). Even when IPV curriculum content is studied during different years, it may not be in a standardized format. Examples in the literature show that most IPV curriculum contents are delivered as single, stand-alone items and are not re-considered during the medical curriculum (43, 63).

When comparing perceived mastery levels with a benchmark, the results are questionable. The results from the present study confirm reports from the literature—though mainly related to professionals—about the low knowledge and awareness level in medical staff, relating misconceptions, and prejudices about IPV (25). In our study, the highest mean IPV score—but still below par—is observed in attitudes. Nevertheless, the current perceived mastery levels can be considered as a baseline to direct future research aiming at improving IPV related education or in view of comparison with medical students and staff in other contexts or geographical settings.

### Limitations

There are four major limitations in this study that could be addressed in future research. First, the study focused on medical students' “perceived” mastery of IPV competences. Though self-efficacy is accepted as a valid precursor of actual behavior (64), alternative approaches could build on video-vignettes to map behavioral proxies of students' mastery. Secondly, we involved students from different medical schools. But this was hardly linked to differences in curriculum implementation or staff competence. Thirdly, 6th-year students were underrepresented. The input from 3rd-year students may reflect their lack of exposure to clinical situations, including intimate partner violence. Finally, female students reported higher self-mastery of IPV. As IPV is more reported in the female population, we did not ask the students if they had an episode of IPV. This could help us to understand if the higher self-reported mastery of female students is related to an episode of IPV.

## Conclusions

Taking into account the present research findings, a need arises to develop an IPV focus in the Mozambican medical school's curricula. Though currently perceived mastery of competencies needed by physicians to deal with IPV of patients is below par, researchers can use the present findings as a baseline for further research fostering the development of IPV competencies. The IPV framework can also guide the development of interventions with a continuous focus underlying knowledge, skills and attitudes components. In the context of follow-up studies, we will focus on online clinical simulations as an avenue to start up related IPV competence development in medical contexts.

### Recommendation for Further Research

Further research is needed to identify the approaches and assessment procedures in the teaching-learning process to present IPV content in Mozambican medical schools. After analyzing the curricula in IPV contents, medical educators can judge the results as to implement an innovative IPV curriculum contents for medical students as designed by the medical schools, or to improve the contents and then implement. It is crucial to evaluate medical students' and medical doctors' perceptions and attitudes toward perpetrators and victims, including their levels of empathy and management skills in dealing with the victims. An evaluation of how the victims of IPV are currently taken care of in different levels of attendance would identify the insufficiencies and omissions that could provide input to guide the introduction of changes in the curriculum. A professional development analysis can help to understand where professional development can contribute to improving medical doctors' competence in delivering IPV curriculum contents and dealing with IPV survivors. Lastly, the instrument developed in the context of this study can be used in other contexts to foster an understanding of IPV-related competencies of medical professionals and eventually the care of IPV victims and conduct a study among practicing physicians to get their perceptions of their competency in dealing with IPV victims.

## Previous Presentations

The authors presented the preliminary results of the first data collection set of 167 students at the AMEE Conference 2017, held in Helsinki, Finland, on August 29, 2017.

## Data Availability

The datasets generated for this study are available on request to the corresponding author.

## Ethics Statement

The protocol was approved by the Institutional Committee of Bioethics and Health linked to the Faculty of Medicine/Central Hospital of Maputo, Mozambique (registration number CIBS FM & MCH/006/2016). All subjects gave written informed consent in accordance with the Declaration of Helsinki.

## Author Contributions

BM, AT, IK, KR, and MV were involved in revising the manuscript critically for important intellectual content, gave final approval of the version to be published, agreed to be accountable for all aspects of the work in ensuring that questions related to the accuracy or integrity of any part of the work are appropriately investigated and resolved and made substantial contributions to conception and design, or acquisition of data, or analysis and interpretation of data.

### Conflict of Interest Statement

The authors declare that the research was conducted in the absence of any commercial or financial relationships that could be construed as a potential conflict of interest.

## References

[1] WHO and PAHO Intimate Partner Violence: Understanding and Addressing Violence Against Women. World Health Organization and Pan American Health Organization (2012). p. 1–12. Retrieved from: https://www.who.int/reproductivehealth/topics/violence/vaw_series/en/

[2] WHO. Health Care for Women Subjected to Intimate Partner Violence or Sexual Violence A Clinical Handbook. World Health Organization (2014). Retrieved from: www.who.int/reproductivehealth

[3] Zacarias AE Women as victims and perpetrators of intimate partner violence (IPV) in Maputo City, Mozambique: occurrence, nature and effects. In: Women as Victims and Perpetrators of Intimate Partner Violence (IPV) in Maputo City, Mozambique: Occurrence, Nature and Effects. (2012). Retrieved from: http://publications.ki.se/xmlui/bitstream/handle/10616/41233/Avhandling_Zacarias.pdf?sequence=1 10.2147/IJWH.S29427

[4] Cruz GV Domingos L Sabune A The characteristics of the violence against women in Mozambique. Health. (2014) 6:1589–601. 10.4236/health.2014.613192

[5] Tjaden P Thoennes N Extent, nature, and consequences of intimate partner violence: findings from the national violence against women survey. Natl Inst Just. (2000) 2000:1–62. 10.1037/e300342003-001

[6] McCloskey LA Boonzaier F Steinbrenner SY Hunter T Determinants of Intimate partner violence in Sub-Saharan Africa: a review of prevention and intervention programs. Partn Abuse. (2016) 7:277–315. 10.1891/1946-6560.7.3.277

[7] Mulawa M Kajula LJ Yamanis TJ Balvanz P Kilonzo MN Maman S. Perpetration and victimization of intimate partner violence among young men and women in Dar es Salaam, Tanzania. J Interpers Viol. (2018) 33:2486–511. 10.1177/0886260515625910 26802044 PMC4956596

[8] Swedo EA Sumner SA Hillis SD Aluzimbi G Apondi R Atuchukwu VO. Prevalence of violence victimization and perpetration among persons aged 13–24 years — Four Sub-Saharan African countries, 2013–2015. MMWR. (2019) 68:350–5. 10.15585/mmwr.mm6815a3 30998666 PMC6476058

[9] Mateus A Representações Sociais de Mulheres sobre Violência Contra a Mulher nas Relações Conjugais na cidade de Maputo, Moçambique (Universidade de Brasília). (2015). Retrieved from: http://repositorio.unb.br/handle/10482/19437

[10] Ministério do Género Criança e Acção Social Plano Nacional de Acção para Prevenção e Combate à Violência contra a Mulher. (2007). Retrieved from: http://forumulher.org.mz/wp-content/uploads/2018/09/Plano-Nac-Prev-Combate-Violencia-Baseada-no-Genero-APROVADO-CM-28.08.2018.pdf

[11] Pathfinder International Multisectoral Responses To Gender-Based Violence in Mozambique. (2015). Retrieved from: www.pathfinder.org

[12] Instituto Nacional de Estatística Mozambique Moçambique Inquérito Demográfico e de Saúde 2011. In: MEASURE DHS, ICF International. (2011). Retrieved from: https://dhsprogram.com/pubs/pdf/FR266/FR266.pdf

[13] Zacarias AE Macassa G Soares JJF Women as perpetrators of IPV: The experience of Mozambique. J Aggress Confl Peace Res. (2012) 4:5–27. 10.1108/17596591211192966

[14] Andersson N Ho-Foster A Mitchell S Scheepers E Goldstein S. Risk factors for domestic physical violence: National cross-sectional household surveys in eight southern African countries. BMC Women Health. (2007) 7:11. 10.1186/1472-6874-7-11 17631689 PMC2042491

[15] Jetha E Lynch C Houry D Rodrigues M Chilundo B Sasser S. Characteristic of victims of family violence seeking care at health centers in Maputo, Mozambique. J Emerg Trauma Shock. (2011) 4:369. 10.4103/0974-2700.83866 21887028 PMC3162707

[16] Colombini M Mayhew S Ali SH Shuib R Watts C I feel it is not enough… Health providers' perspectives on services for victims of intimate partner violence in Malaysia. BMC Health Serv Res. (2013) 13:65 10.1186/1472-6963-13-65 23419141 PMC3582493

[17] Banyard VL Demers JM Cohn ES Edwards KM Moynihan MM Walsh WA Academic correlates of unwanted sexual contact, intercourse, stalking, and intimate partner violence: an understudied but important consequence for college students. J Interpers Viol. (2017) 2017:22 10.1177/0886260517715022 29294800

[18] Hamberger LK Ambuel B Guse CE Racial differences in battered women's experiences and preferences for treatment from physicians. J Fam Viol. (2007) 22:259–65. 10.1007/s10896-007-9071-5

[19] Roelens K Verstraelen H Van Egmond K Temmerman M. A knowledge, attitudes, and practice survey among obstetrician-gynaecologists on intimate partner violence in Flanders, Belgium. BMC Public Health. (2006) 6:238. 10.1186/1471-2458-6-238 17002786 PMC1635712

[20] Van Den Ameele S Keygnaert I Rachidi A Roelens K Temmerman M. The role of the healthcare sector in the prevention of sexual violence against sub-Saharan transmigrants in Morocco: a study of knowledge, attitudes and practices of healthcare workers. BMC Health Serv Res. (2013) 13:77. 10.1186/1472-6963-13-77 23442386 PMC3608151

[21] Tivane AF Violência doméstica Contra os Homens Perpetrada pelas suas Mulheres no Contexto Conjugal no Bairro da Mafalala (Eduardo Mondlane University). (2015). Retrieved from: www.saber.ac.mz

[22] Swailes AL Lehman EB McCall-Hosenfeld JS. Intimate partner violence discussions in the healthcare setting: a cross-sectional study. Prev Med Rep. (2017) 8:215–20. 10.1016/j.pmedr.2017.10.017 29159016 PMC5683665

[23] Ramsay J Rutterford C Gregory A Dunne D Eldridge S Sharp D. Domestic violence: knowledge, attitudes, and clinical practice of selected UK primary healthcare clinicians. Br J Gen Pract. (2012) 647–55. 10.3399/bjgp12X654623 22947586 PMC3426604

[24] Colarossi L Breitbart V Betancourt G. Barriers to screening for intimate partner violence: a mixed-methods study of providers in family planning clinics. Perspect Sex Reproduc Health. (2010) 42:236–43. 10.1363/4223610 21126299

[25] Tripp M Short LM Gadomski AM Lewis C Wolff D. Changes in health care providers' knowledge, attitudes, beliefs, and behaviors regarding domestic violence, following a multifaceted intervention. Academ Med. (2004) 76:1045–52. 10.1097/00001888-200110000-00015 11597847

[26] Almutairi GD Alrashidi MR Almerri AT Kamel MI El-Shazly M How to screen for domestic violence against women in primary health care centers. Alexandr J Med. (2013) 49:89–94. 10.1016/j.ajme.2012.07.004

[27] Varjavand N Cohen DG Novack DH. An assessment of residents' abilities to detect and manage domestic violence. J Gen Intern Med. (2002) 17:465–8. 10.1046/j.1525-1497.2002.10404.x 12133162 PMC1495067

[28] Frank E Elon L Saltzman LE Houry D McMahon P Doyle J. Clinical and personal intimate partner violence training experiences of U.S. Medical Students. J Women Health. (2006) 15:1071–9. 10.1089/jwh.2006.15.1071 17125426

[29] Lawoko S Sanz S Helström L Castren M. Screening for intimate partner violence against women in healthcare sweden: prevalence and determinants. ISRN Nurs. (2011) 2011:1–7. 10.5402/2011/510692 22254143 PMC3255304

[30] Fawole O Van Wyk JM Adejimi A Training on prevention of violence against women in the medical curriculum at the University of Ibadan, Nigeria. Afr J Health Profess Edu. (2013) 5:75 10.7196/ajhpe.222

[31] Aluko OE Beck KH Howard DE. Medical students' beliefs about screening for intimate partner violence: a qualitative study. Health Promot Pract. (2015) 16:540–9. 10.1177/1524839915571183 25663054

[32] American Academy of Family Physicians Recommended Curriculum Guidelines for Family Medicine Residents–Conditions of the Eye. (2013). Retrieved from: http://www.aafp.org/medical-school-residency/program-directors/curriculum.html

[33] Connor PD Nouer SS Mackey SN Banet MS Tipton NG. Intimate partner violence education for medical students: toward a comprehensive curriculum revision. Southern Med J. (2012) 105:211–5. 10.1097/SMJ.0b013e31824f8b01 22475671

[34] Schraiber LB Latorre M do RDO França I Segri NJ D'Oliveira AFPL. Validity of the WHO VAW study instrument for estimating gender-based violence against women. Rev Saude Publ. (2010) 44:658–66. 10.1590/S0034-89102010000400009 20676557

[35] Short LM Johnson D Osattin A. Recommended components of health care provider training programs on intimate partner violence. Am J PrevMed. (1998) 14:283–8. 10.1016/S0749-3797(98)00007-5 9635072

[36] Kelmendi K Violence against women: methodological and ethical issues. Psychology. (2013) 4:559–65. 10.4236/psych.2013.47080

[37] Nybergh L Taft C Krantz G. Psychometric properties of the WHO violence against Women instrument in a female population-based sample in Sweden: a cross-sectional survey. BMJ Open. (2013) 3:e002053. 10.1136/bmjopen-2012-002053 23793692 PMC3664346

[38] Roelens K Verstraelen H Temmerman M. Intimate partner violence. The gynaecologist's perspective. Facts Views Vis Obgyn. (2009) 1:88–98. 25478074 PMC4251275

[39] Short LM PREMIS: Measuring IPV Knowledge, Attitudes and Practices of Health Care Practitioners. (n.d). Retrieved from: https://www.futureswithoutviolence.org/userfiles/file/HealthCare/LynnShort.pdf

[40] Kamimura A Al-Obaydi S Nguyen H Trinh HN Mo W Doan P. Intimate partner violence education for medical students in the USA, Vietnam and China. Public Health. (2015) 129:1452–8. 10.1016/j.puhe.2015.04.022 26047798

[41] Valpied J Aprico K Clewett J Hegarty K. Are future doctors taught to respond to intimate partner violence? a study of australian medical schools. J Interpers Viol. (2017) 32:2419–32. 10.1177/0886260515592616 26185216

[42] Carlson BE Worden AP Attitudes and beliefs about domestic violence: results of a public opinion survey. J Interpers Viol. (2005) 20:1197–218. 10.1177/0886260505278530 16162486

[43] Alpert EJ Tonkin AE Seeherman AM Holtz HA. Family violence curricula in U.S. Medical schools. Am J Prev Med. (1998) 14:273–82. 10.1016/S0749-3797(98)00008-7 9635071

[44] Hill JR. Teaching about family violence: a proposed model curriculum. Teach Learn Med. (2005) 17:169–78. 10.1207/s15328015tlm1702_12 15833728

[45] Joyce BL Jung D Lucia VC Kavanagh M Afonso N Developing medical student competence in intimate partner violence: a National priority. Med Sci Educ. (2015) 25:229–32. 10.1007/s40670-015-0144-4

[46] Tufts KA Clements PT Karlowicz KA. Integrating intimate partner violence content across curricula: developing a new generation of Nurse Educators. Nurse Educ Today. (2009) 29:40–7. 10.1016/j.nedt.2008.06.005 18692280

[47] Hamberger LK. Preparing the next generation of physicians: medical school and residency-based intimate partner violence curriculum and evaluation. Trauma Viol Abuse. (2007) 8:214–25. 10.1177/1524838007301163 17545575

[48] Carlson M Kamimura A Al-Obaydi S Trinh HN Franchek-Roa K. Background and clinical knowledge of intimate partner violence: a study of primary care residents and medical students at a United States medical school. Health Equ. (2017) 1:77–82. 10.1089/heq.2017.0008 30283836 PMC6071885

[49] Assessment of Competency Benchmarks Work Group Assessment of Competency Benchmarks Work Group: A Developmental Model for the Defining and Measuring Competence in Professional Psychology June 2007. (2007).

[50] Folberg R Antonioli DA Alexander CB. Competency-based residency training in pathology: challenges and opportunities. Hum Pathol. (2002) 33:3–6. 10.1053/hupa.2002.30226 11823968

[51] Chacko T Moving toward competency-based education: challenges and the way forward. Arch Med Health Sci. (2014) 2:247 10.4103/2321-4848.144365

[52] Zimmerman BJ Dibenedetto MK Mastery learning and assessment: implications for students and teachers in an era of high-stakes testing. Psychol Schools. (2008) 45:206–16. 10.1002/pits.20291

[53] Temmerman M. Research priorities to address violence against women and girls. Lancet. (2015) 385:e38–40. 10.1016/S0140-6736(14)61840-7 25467581

[54] Nulty DD The adequacy of response rates to online and paper surveys: what can be done? Assess Eval Higher Educ. (2008) 33:301–14. 10.1080/02602930701293231

[55] The University of Texas Response Rates. Encyclopedia of Survey Research Methods. (2008). p. 759–62. Retrieved from: http://ctl.utexas.edu/sites/default/files/response_rates.pdf

[56] Bandura A Guide for constructing self-efficacy scales. In: Self-Efficacy Beliefs of Adolescents. (2006). p. 307–37. Retrieved from: https://www.uky.edu/~eushe2/Bandura/BanduraGuide2006.pdf

[57] Hu LT Bentler PM Cutoff criteria for fit indexes in covariance structure analysis: conventional criteria versus new alternatives. Struc Equ Model. (1999) 6:1–55. 10.1080/10705519909540118

[58] Jackson DL Gillaspy JA Purc-Stephenson R. Reporting practices in confirmatory factor analysis: an overview and some recommendations. Psychol Methods. (2009) 14:6–23. 10.1037/a0014694 19271845

[59] Baguley T. Standardized or simple effect size: what should be reported? Br J Psychol. (2009) 100:603–17. 10.1348/000712608X377117 19017432

[60] Verdonk P Mans LJL Lagro-Janssen ALM. Integrating gender into a basic medical curriculum. Med Educ. (2005) 39:1118–25. 10.1111/j.1365-2929.2005.02318.x 16262807

[61] Zaher E Keogh K Ratnapalan S. Effect of domestic violence training: systematic review of randomized controlled trials. Can Fam Phys. (2014) 60:618–24. 10.1037/e529382014-198 25022633 PMC4096259

[62] UN Women Handbook for National Action Plans on Violence Against Women. (2012). Retrieved from: http://www.endvawnow.org/uploads/browser/files/handbook-for-nap-on-vaw.pdf

[63] Rivas C Ramsay J Sadowski L Davidson L Dunne D Eldridge S Advocacy interventions to reduce or eliminate violence and promote the physical and psychosocial wellbeing of women who experience intimate partner abuse: a systematic review. Campb System Rev. (2016) 2016:2 10.4073/csr.2016.2

[64] Ajzen I Perceived behavioral control, self-efficacy, locus of control, and the theory of planned behavior. J Appl Soc Psychol. (2002) 32:665–83. 10.1111/j.1559-1816.2002.tb00236.x

