# Supplementary material for: Gaps in Medical Students' Competencies to Deal With Intimate Partner Violence in Key Mozambican Medical Schools
Source: Front Public Health. 2019 Jul 24;7:204. doi: 10.3389/fpubh.2019.00204 (PMC6667801; doi:10.3389/fpubh.2019.00204)
Supplement: Supplementary file 1 [file Data_Sheet_1.pdf]

Appendix 1. Framework of knowledge, attitudes, and skills on IPV

**Knowledge**	1. Demonstrate knowledge and understand the magnitude of the problem:<br>– intimate partner violence as a public health problem,<br>– the cycle of violence in relationships and families,<br>– the medical and mental health implications for victims and their families,<br>– the unique role that health care providers can play,<br>– the barriers to providing services to victims.<br>2. Demonstrate knowledge and understand uncommon circumstances:<br>– abuse during pregnancy,<br>– substance use and abuse,<br>– IPV in same-sex partners,<br>– HIV risks for victims of IPV,<br>– the legal concerns of immigrants and refugees,<br>– dealing with the health care provider who has experienced or perpetrated IPV.<br>3. Demonstrate knowledge and understand life-span issues:<br>– the relationship between child abuse and neglect,<br>– the short- and long-term outcomes for children who witness violence in their homes,<br>– elder abuse by previously abused children,<br>– the cumulative effects of multiple victimizations,<br>– other issues related to the types of violence encountered in different parts of the life cycle.<br>4. Demonstrate knowledge and understand legal options and reporting requirements:<br>– be aware of the requirements for reporting intimate partner violence,<br>– be aware of the role in providing epidemiologic information on the numbers of survivors and the patterns of abuse.
**Skills**	**1.** Identification; be able to**:**<br>– screen universally,<br>– ask questions appropriately to recognize survivors of abuse,<br>– obtain a history of abuse,<br>– determine the patient's immediate risk of danger,<br>– assess the patient's mental health needs,<br>– identify types of abuse against the patient or other family members through a physical and psychological examand<br>– identify general and specific signs and symptoms of distress in victims of abuse.<br><br>2. Reporting; be able to<br>– develop body injury maps,<br>– manage abuse appraisal screens.
**Attitudes**	1. Treatment and intervention:<br>– assure patient confidentiality,<br>– be understanding, sensitive and patient,<br>– provide support in a respectful, nonjudgmental manner.<br>2. Safety:<br>– effectively intervene without placing the patient (or provider) in greater danger: interview patients in private, maintain separation from an accompanying partner, assure confidentiality and provide privacy and anonymity to protect the victim's safety.<br>3. Availability:<br>- be accessible for future contacts by the survivors.

Appendix 2 IPV self-efficacy perspective scale

KNOWLEDGE

Understand the nature of the community support that can be given to IPV survivors.

Understand the nature of successful treatment options for perpetrators of IPV.

Understand the legal requirements to inform police about cases of IPV.

Being able to identify signs and symptoms of IPV in cases, presented by survivors or perpetrators.

Understand the epidemiology of IPV as it is related to race, gender and sexuality and substance abuse.

Being able to identify the predispositions and characteristics of survivors or perpetrators.

Know about the psychological competence needed to treat IPV survivors safely and with empathy.

Being able to define intimate partner violence.

SKILLS

Being able to document in a correct way cases of IPV.

Being able to pay attention to one's body language when interacting with a perpetrator.

Being able to develop a medical history of IPV in an empathic and non-judgmental way.

Being able to develop a detailed medical history of a suspected IPV survivor.

Being able to develop a strategy to interact with the local community when IPC cases have been identified (e.g., working with social workers, police ...).

Being able to develop a detailed medical history of a patient, without prior knowledge about his/her IPV status.

See the need to follow up survivors of IPV and see whether they feel safe at home.

ATTITUDES

Overcome the stereotypes around IPV (e.g., higher prevalence in some ethnic groups).

Understand the profound impact of IPV on survivors and the immediate and future impact on their children.

Understand the limitations in confidentiality when it comes to IPV.

Appreciate the multidisciplinary nature of help to be given to survivors of IPV.

Understand the barriers for the survivors to admit they need help and to seek for help.

Understand the barriers for medical doctors to identify survivors of IPV.

Appendix 3 Results of the confirmatory factor analysis (CFA) of the IPV perceived mastery scale

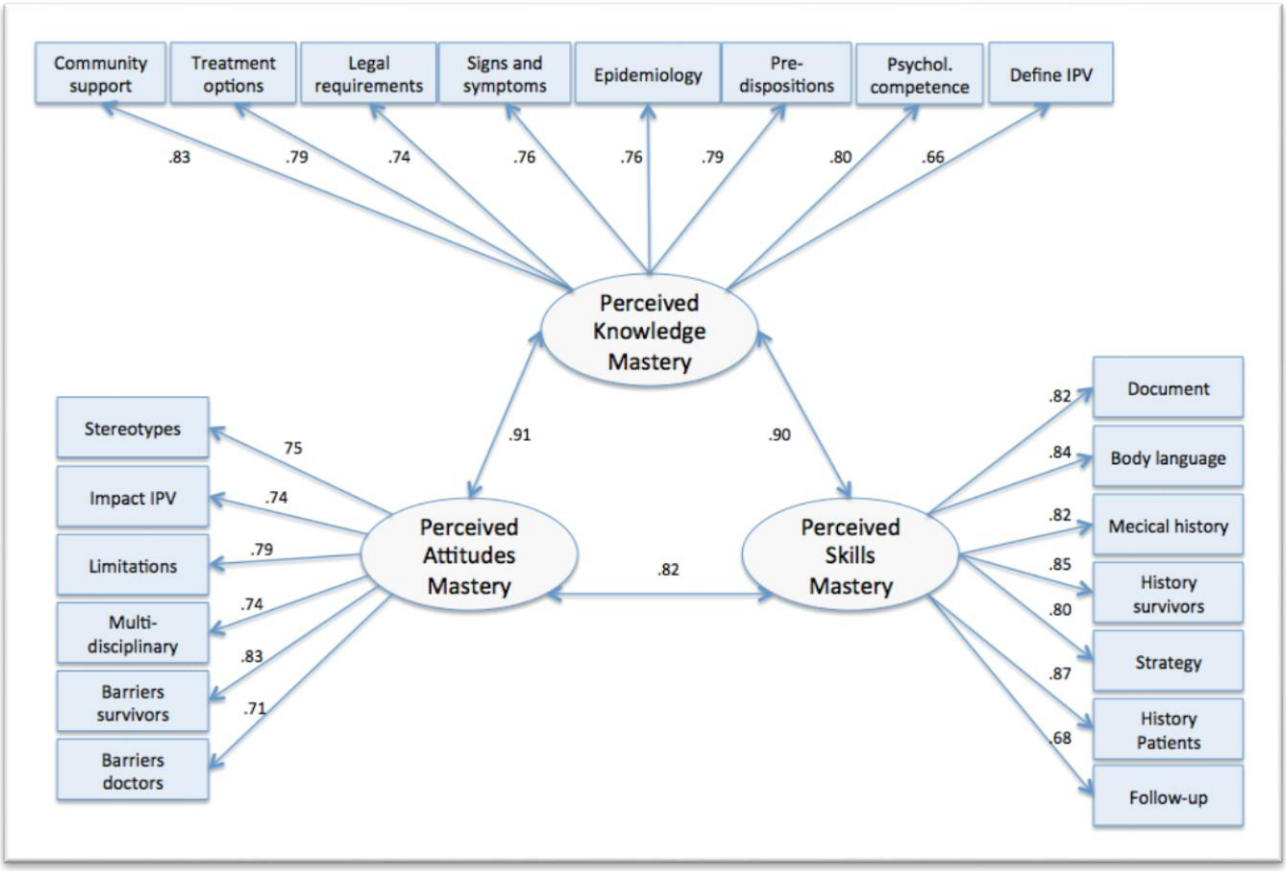

Appendix 4. Medical students' IPV self-efficacy perspective questionnaire

Your candid responses on the following survey will greatly assist us in our attempt to improve physicians' recognition and management of intimate partner violence-related injuries and illnesses. Please give us your first, instinctive answer, even if you do not think it is "politically correct." Do not try to think about what your answers "should" be. All responses will be coded by an identifying number only, kept confidential, and analyzed at group level so that no personal information is revealed. We used a self-efficacy perspective rating scale 0 to 100. In each section, we will explain the meaning of this scale. Each section will address issues related to your medical training and how it has introduced to you, or helped you to tackle Intimate Partner Violence in a medical setting. Some questions may look similar to others. However, we ask you to answer all questions, to ensure the reliability of your assessment.

Thank you for taking the time - estimated at 25 minutes - for this survey.

Today's date: ____/ ____/ ____

Section I: Background information

1. Your Age: ________________________

2. Sex: [ ] Male [ ] Female

3. Year in medical school __________ Year

Section II: Your curriculum

1. Have you ever received any formal training on intimate partner violence?<br>**If No,** proceed with question 7	[ ] Yes	[ ] No
	**If Yes**,<br>2. What type of training set up?	[ ] Medical school`<br>[ ] Workshop<br>[ ] Lectures<br>[ ] Others: ___________________________
	**If medical school**? Answer the next questions.<br>**If not**, proceed to Question 8.<br>3. In which year you had the training.	____________________

Section III: Your medical training about Intimate Partner Violence

The way you respond is easy. You give us a number between 0 and 100. Rate your degree of confidence you can carry out the competence related to IPV knowledge, skills and attitudes from 0 to 100 using the scale given below:

0	10	20	30	40	50	60	70	80	90	100
Cannot do at all					Moderately certain can do					Highly certain can do

**KNOWLEDGE**	
Understand the nature of the community support that can be given to IPV survivors.	
Understand the nature of successful treatment options for perpetrators of IPV.	
Understand the legal requirements to inform police about cases of IPV.	
Being able to identify signs and symptoms of IPV in cases, presented by survivors or perpetrators.	
Understand the epidemiology of IPV as it is related to race, gender, sexuality, and substance abuse.	
Being able to identify the predispositions and characteristics of survivors or perpetrators.	
Know about the psychological competence needed to treat IPV survivors safely and with empathy.	

**SKILLS**	
Being able to document in a correct way cases of IPV.	
Being able to pay attention to one's body language when interacting with a perpetrator.	
Being able to develop a medical history of IPV in an empathic and non-judgmental way.	
Being able to develop a detailed medical history of a suspected IPV survivor.	
Being able to develop a strategy to interact with the local community when IPC cases have been identified (e.g., working with social workers, police, ...).	
Being able to develop a detailed medical history of a patient, without prior knowledge about his/her IPV status.	
See the need to follow up survivors of IPV and see whether they feel safe at home.	

**ATTITUDES**	
Overcome the stereotypes around IPV (e.g., higher prevalence in some ethnic groups).	
Understand the profound impact of IPV on survivors and the immediate and future impact on their children.	
Understand the limitations in confidentiality when it comes to IPV.	
Appreciate the multidisciplinary nature of help to be given to survivors of IPV.	
Understand the barriers for the survivors to admit they need help and to seek for help.	
Understand the barriers for medical doctors to identify survivors of IPV.	

Do you think the above list is incomplete? If this is the case, add some suggestions below:

- ____
- ____
- ____

Thank you very much for your time.
